# Durum wheat diversity for heat stress tolerance during inflorescence emergence is correlated to *TdHSP101C* expression in early developmental stages

**DOI:** 10.1371/journal.pone.0190085

**Published:** 2017-12-28

**Authors:** Miguel Bento, Sónia Gomes Pereira, Wanda Viegas, Manuela Silva

**Affiliations:** Linking Landscape, Environment, Agriculture and Food (LEAF), Instituto Superior de Agronomia, Universidade de Lisboa, Lisboa, Portugal; Huazhong University of Science and Technology, CHINA

## Abstract

The predicted world population increase along with climate changes threatens sustainable agricultural supply in the coming decades. It is therefore vital to understand crops diversity associated to abiotic stress response. Heat stress is considered one of the major constrains on crops productivity thus it is essential to develop new approaches for a precocious and rigorous evaluation of varietal diversity regarding heat tolerance. Plant cell membrane thermostability (CMS) is a widely used method for wheat thermotolerance assessment although its limitations require complementary solutions. In this work we used CMS assay and explored *TdHSP101C* genes as an additional tool for durum wheat screening. Genomic and transcriptomic analyses of *TdHSP101C* genes were performed in varieties with contrasting CMS results and further correlated with heat stress tolerance during fertilization and seed development. Although the durum wheat varieties studied presented a very high homology on *TdHSP101C* genes (>99%) the transcriptomic assessment allowed the discrimination between varieties with good CMS results and its correlation with differential impacts of heat treatment during inflorescence emergence and seed development on grain yield. The evidences here reported indicate that *TdHSP101C* transcription levels induced by heat stress in fully expanded leaves may be a promising complementary screening tool to discriminate between durum wheat varieties identified as thermotolerant through CMS.

## Introduction

Cereals are essential in human and domestic animal nutrition and constitute over 50% of crop production worldwide. Cereal crops are however expected to suffer marked effects of climate changes namely resulting from high temperature stress during grain filling [1]. Durum wheat (*Triticum durum* Desf.) can be one of the most affected cereals since it is mainly produced in Southern European countries (Italy, France and Greece) and Canada, where the predictable temperature increases will drastically disturb its productivity [2, 3]. With the projected world population increase in the coming years and the consequently higher demand of agricultural products, it is now more than ever urgent to better understand crops diversity associated to thermal stress tolerance.

High temperature stress has a wide range of effects on plants in terms of physiology, biochemistry and gene regulation pathways. Plants respond to heat stress in two distinct phases: the first is based on the intrinsic tolerance to high temperatures induced damage–basal thermotolerance; and the second phase involves resources mobilization and gene expression changes to cope with heat stress related injury–acquired thermotolerance (reviewed in [4]). High temperature stress occurring during reproductive development can be particularly detrimental affecting plant fertility and yield [5]. Due to complex physiological and developmental events pollen development and fertilization are often considered the weak-links in heat stress tolerance [6]. Though, during wheat endosperm development (grain filling) heat stress is also problematic since it can significantly modify grain protein content affecting overall quality and yield [7]. The search of methods allowing a precocious evaluation of genotypes tolerant to heat stress namely at the inflorescence emergence or during grain development has therefore been a major goal for breeders.

Thermotolerance in plants is the ability to cope with high temperatures, enabling metabolic activity and growth [8]. Temperature increase usually leads to augmented cellular entropy and cellular membranes disruption. The resulting increase in membranes fluidity [4, 9] with the consequent leakage of ions and other electrolytes to the extracellular medium can be measured and used as an indirect quantification method of cell damage [9, 10]. Values of plant cell membrane thermostability (CMS) on young seedlings correlate well with wheat adult plants thermotolerance and grain yield performance under stress conditions [9]. Therefore, CMS is a widely used method to distinguish between thermotolerant and sensitive wheat varieties [11].

Heat stress tolerance is a well documented polygenic trait as plants under stress normally reveal a decrease in overall protein synthesis, associated to marked increases in heat shock protein (HSP) gene expression, augmented phytohormones production, antioxidants and other protective molecules [3]. When plants experience heat stress, the synthesis and accumulation of HSPs occur extremely fast and intensively, representing one of the most important adaptive strategy to overcome high temperature deleterious effects [4]. Generally, HSPs are classified into five groups distinguished by molecular weights: HSP100, HSP90, HSP70, HSP60, and small HSPs with 15–30 kDa. The majority of HSPs are molecular chaperones involved in protein stabilization and signal transduction during heat stress (reviewed in [4]).

In the last years much attention was paid to HSP101 due to the discovery of its major role in basal and acquired thermotolerance [12, 13]. Evidences obtained in *Arabidopsis* also suggest that HSP101 biosynthesis yields substantial fitness benefit under normal growth conditions [14]. Among durum wheat varieties, *HSP101* differential constitutive expression seems to be related with distinct strategies to cope with abiotic stress [15]. Recently, genetically based variation on *HSP101* expression and in plant thermotolerance was associated with the geographical origin of the population and local climate [16]. Additionally, it has been documented the involvement of HSP101 in protein synthesis regulation [17–19]. In *T*. *durum* two different HSP101 isoforms were identified (TdHSP101B and TdHSP101C) each with A and B forms distinguished by characteristic single nucleotide polymorphisms (SNPs) at gene level. The *TdHSP101B* and *TdHSP101C* isoforms have extremely different transcription levels under heat stress conditions. Moreover, it was suggested the existence of different roles between isoforms, with TdHSP101C (A and B forms) being specially associated with durum wheat acquired thermotolerance [20].

In this work we intent to use CMS seedling screening to identify *T*. *durum* varieties with contrasting heat stress tolerance patterns. We further evaluate how *TdHSP101C* genomic or transcriptomic differences could be correlated with distinct degrees of thermotolerance in key plant developmental phases.

## Materials and methods

### Plant material

Seven durum wheat varieties (*Triticum durum*, 2n = 4x = 28, AABB) from the European database of plant varieties with different geographic proveniences were used in this study: three Portuguese varieties—Celta, Hélvio and Marialva; three Italian varieties–Saragolla, Severo and Simeto; and one Greek variety—DonDuro. All seeds were germinated in Petri dishes in growth chamber with controlled conditions (cycle 16h light/25°C and 8h dark/20°C) and transferred to perlite for thermotolerance assessment or to soil pots until further use. After six weeks soil pots were transferred to greenhouse conditions.

### Thermotolerance screening through cell membrane thermostability (CMS) evaluation

To evaluate the basal thermotolerance ten-day-old seedlings were maintained in growth chamber with the described conditions (cycle 16h light / 25°C and 8h dark / 20°C) with 80% relative humidity and 250 μmolm^-2^s^-1^ photosynthetic photon flux density (PPFD). To assess acquired thermotolerance, accordingly to the procedure described in [21], seedlings were exposed to a temperature increase of 3.5°C h^-1^ (starting immediately after the night period of the ninth day) until reached 34°C, temperature that was maintained for 24 h. Immediately after, cell membrane stability was measured as described by [9] in triplicates for control (C) and treatments (T) in 3.5 cm long leaf segments excised from all seedlings. Leaf segments were firstly rinsed in distilled water and placed in closed tubes with 1 ml of distilled water in a water bath at 52°C for 1 h whereas control replicates were kept at 10°C. Afterwards 9 ml of distilled water were added to each tube and samples were incubated at 10°C for 24 h. After reaching room temperature the solution conductivity (C1, T1) was measured. All tubes were then autoclaved at 121°C (1.5 MPa) for 15 min and samples conductivity (C2, T2) was measured again. CMS (%) was calculated as [(1−T1/T2)/(1−C1/C2)]×100 [8] and the results obtained were compared by t-test and One-way ANOVA with Tukey’s multiple comparison test using GraphPad Prism (GraphPad Software, Inc.).

### Evaluation of heat stress impact on grain productivity

For the evaluation of heat stress effects on grain yield two distinct and independent one week high temperature treatments (HTS1 and HTS2) were performed in growth chamber in at least 10 plants of Celta, Hélvio and Marialva varieties. All plants were constantly monitored to identify the desired development stages and were then transferred to growth chambers with 8 h dark at 20°C and 16 h light cycle (temperature dependable of selected treatment) with 80% relative humidity and 250 μmolm^-2^s^-1^ PPFD. Pots were watered daily to ensure a level between 70–75% of soil maximum water holding capacity. HST1 was performed during inflorescence emergence, starting immediately after first awns appearance (Zadoks decimal code 49—First awns visible, [22]), adapting the procedure described in [7]. In such treatment plants were subjected to a daily progressive temperature increase from 20°C to 34°C (2.33°C h^-1^, HST1) or from 20°C to 25°C (0.83°C h^-1^, control) initiating immediately after the dark period. Top temperatures were maintained during 4h and then progressively decreased inverting the previously described temperature rates until dark conditions (8 h at 20°C). HST2 was implemented ten days after anthesis beginning (Zadoks decimal code 61—Anthesis complete, [22]), following the procedure referred in [23]. For HST2, plants were exposed to one week treatment similar to the one previously described for HTS1 but with 40°C top temperature (increase rate of 3.33°C h^-1^). After HST1 and HST2 plants were maintained in greenhouse until seed maturation and grain yield of control and heat-treated plants was comparatively evaluated. Grain yield was assessed always in spikes from the primary tiller through the quantification of the number of grains per spike and the average weight of 10 grains randomly selected (10 sets of 10 grains were analyzed for each variety and condition). Means and standard errors (SE) were calculated and used to perform t-test and One-way ANOVA with Tukey’s multiple comparison test using GraphPad Prism (GraphPad Software, Inc.).

### DNA isolation, PCR amplification, cloning and sequencing

DNA was isolated from fresh young leaves using Citogene^®^ DNA Purification Kit (Citomed). PCR amplification of *TdHSP101C* coding sequence targeting the protein C-terminal region (including the AAA+ and ClpB_D2-small conserved domains) was performed using primers previously designed by [21]. PCR reactions with 50 μl were prepared with: 20 mM Tris–HCl (pH 8.4), 50 mM KCl, 1.5 mM MgCl2, 0.25 mM dNTP’s, 1 mM each primer (forward 5’-GTTGGACAGTATGAGGCCGT-3’; reverse 5’-CATTTCACCCCCAATTCAACAG-3’), 0.5 U *Taq* polymerase and 25 ng DNA template. The following program was used: 3 min at 95°C; 30 cycles of 30 s at 95°C, 30 s at 60°C, 45 s at 72°C; termination by 5 min of final extension at 72°C. PCR products were separated through electrophoresis in 1.7% agarose gels stained with ethidium bromide and photographed using Bio-Rad GEL DOC 2000. Selected bands were gel isolated and purified using PureLink^®^ Quick Gel Extraction Kit (Invitrogen) and cloned using TA Cloning^®^ Kit (Invitrogen). Selected colonies were grown overnight in 5 ml LB broth containing 100 μg/ml ampicillin, plasmids were isolated using NZYMiniprep^®^ kit (Nzytech) and finally sequenced through Sanger Sequencing.

### *In silico* sequence analysis

DNA sequences were edited using BioEdit sequence alignment editor (version 7.1.6.0), compared with public databases using NCBI Blastn with algorithm parameters set for default values (http://blast.ncbi.nlm.nih.gov/Blast.cgi) and multiple sequence alignments were obtained with ClustalW [24]. Augustus software (http://bioinf.uni-greifswald.de/augustus/) was used to predict exon-intron structure and the resulting peptide sequences. The results obtained were confirmed with Eucaryotic GeneMark (http://exon.gatech.edu/eukhmm.cgi) and Expasy Translate Tool (http://web.expasy.org/translate/). Haplotype median joining networks were obtained using NETWORK 4.6.1.2 (fluxus-engineering.com, [25]).

### Evaluation of *TdHSP101C* transcription levels

*TdHSP101C* transcriptomic analysis was performed in one month old plants of Celta, Hélvio and Marialva varieties. For heat stress treatment, plants were submitted during 4 h to 34°C (3°C h^-1^ increase from 20 to 34°C starting immediately after dark period) whereas untreated control plants were exposed during 4 h to 25°C (1°C h^-1^ increase from 20 to 25°C) as described in [20] with minor modifications. Immediately after, leaves were collected, frozen in liquid nitrogen and stored separately at -80°C until used for RNA extraction. Total RNA was extracted with RNAqueous Isolation Kit (Ambion). After verifying RNA concentration and integrity, 1 μg of total RNA was used to perform RQ1 RNase-Free DNase digestion (Promega) and first strand cDNA synthesis using iScriptTM cDNA Synthesis Kit (BIO-RAD). *TdHSP101C* transcription levels were analyzed by quantitative real-time-PCR (qRT-PCR) with BIO-RAD IQ5 Multicolor Real-Time PCR detection System in three replicates for control and stress treatments.

qRT-PCR with primers specific for wheat *TdHSP101C* gene previously designed by [21] (forward 5’-CGAGAACTCCACGGTGTACATC-3’; reverse 5’- TGCTTGTCGACGCCATAGG-3’) as well as for *Actin1* gene used as internal reference gene (forward 5’-ACAATTTCCCGTTCGGCAGTG-3’; reverse 5’- ACATGCCATCCTTCGTCTTGAC-3’) were performed with the SsoFastTM EvaGreen^®^ Supermix (BIO-RAD). Each 20 μl PCR mix containing 20 ng of first strand cDNA, 10 μl SsoFast EvaGreen supermix and forward and reverse primers (500 nM each) were amplified for 40 cycles (95°C-30 s, 40 cycles of 95°C-10 s, 60°C-10 s, and 72°C-10 s). Melt curves were analyzed to ensure amplification of single products as well as to estimate their melting temperatures and PCR products were separated by electrophoresis in 1.5% agarose gel stained with ethidium bromide. Quantification analysis was performed through the ΔΔCt method using threshold cycles (Ct) equilibrated with mean *Actin1* to calculate ΔCt (ΔCt = Ct of interest–mean *Actin1* Ct). *TdHSP101C* expression levels were analyzed calculating ΔΔCt (ΔΔCt = ΔCt stress–mean ΔCt control), further used to estimate mean fold change (2^(-ΔΔCt) ± standard errors between treatments). The quantitative *TdHSP101C* transcription levels evaluation for each variety was performed through a pair wise comparative analysis between control and heat stress expression levels. The comparative analysis between distinct varieties expression levels was normalized against Celta transcript level. Means and standard errors (SE) were calculated and used to compute t-test and One-way ANOVA with Tukey’s multiple comparison test using GraphPad Prism (GraphPad Software, Inc.).

## Results

### Early CMS screening revealed distinct thermotolerance abilities

**Fig 1:**
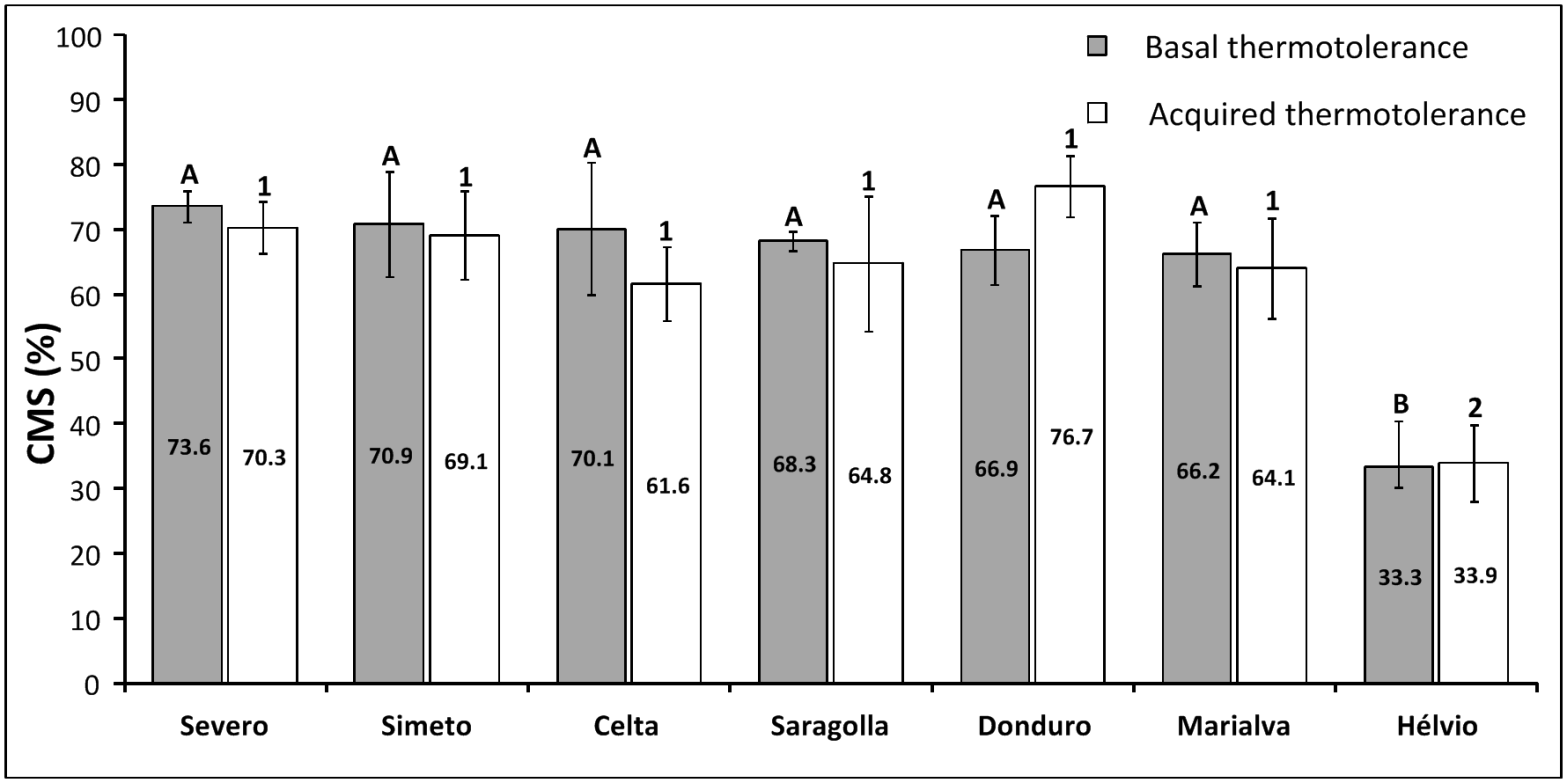
Cell membrane thermostability assay. Basal and acquired thermotolerance values of seven durum wheat varieties estimated by cell membrane stability (CMS) assay. Different letters or numbers indicate varieties with basal or acquired thermotolerance significantly different (p<0.05) as identified by one-way ANOVA.

The comparison between basal and acquired thermotolerance values obtained by CMS assay for each variety tested did not show significant differences (t-test, p<0.05). However, the comparison between distinct varieties through early CMS evaluation disclosed one variety with a significant distinct basal and acquired thermotolerance in comparison to the remaining ones (Fig 1). Therefore, the variety Hélvio was considered sensitive whereas all the others were scored as tolerant in comparison with previous reports using the same methodology to assess durum wheat thermotolerance [21,26]. These results were supported by One-way ANOVA and Tukey’s multiple comparison test (p<0.05) for both basal and acquired thermotolerance (Fig 1).

To further explore the correlation of CMS evaluation values with heat stress performance at different developmental stages we selected Portuguese varieties with different heat stress tolerance levels: Celta and Marialva from the group showing high CMS values and Hélvio with significant lower thermotolerance.

### Durum wheat varieties with high CMS values are differentially affected by heat stress at distinct reproductive phases

To evaluate the impact of the two heat treatments performed–HST1 during inflorescence emergence stage (34°C) and HST2 during grain filling stage (40°C)–two yield parameters were evaluated: number of grains per spike and average grain weight (Table 1). In control conditions the number of grains per spike was highly variable between varieties, Hélvio presented the lowest average number of seeds (~11) and Marialva the highest (~26) (Table 1). Regarding this grain yield parameter, none of the varieties studied showed significant differences (t-test, p>0.05), neither between control and HST1 plants nor between control and HST2 plants. Contrastingly, the average grain weight of plants grown at control conditions was very similar between varieties while marked differences existed in heat stress responses. Comparison of average grain weight between control and HST1 plants revealed that only Marialva was significantly affected, leading to a ~18% reduction on grain weight. On the other hand, significant reductions induced by HST2 were detected on average grain weight (p<0.001) of all three varieties studied (Celta ~25%; Hélvio ~36% and Marialva ~14%) (Table 1).

**Table 1:** Impact of heat stress treatment at inflorescence emergence stage (HST1) and at grain filling stage (HST2) on grain yield of Portuguese durum wheat varieties.

	Variety	N. of grains per spike			Average grains weight (g)			
		Control	Stress	Significant	Control	Stress	Significant	Variation (%))
**HST1**	Celta	20.9	21.7	ns	0.545	0.499	ns	**-8.4**
	Hélvio	10.7	11.9	ns	0.582	0.571	ns	**-1.9**
	Marialva	26.4	27.8	ns	0.544	0.445	***	**-18.2**
**HST2**	Celta	20.9	19.1	ns	0.545	0.409	***	**-25.0**
	Hélvio	10.7	16.7	ns	0.582	0.374	***	**-35.7**
	Marialva	26.4	26.9	ns	0.544	0.466	***	**-14.3**

ns—not significant (p value >0.05);

*** significant differences between control and HTS1 or HTS2 (p<0.001) identified by t-test.

The effect of HST2 on the average weight is more detrimental than HST1 for Celta and Hélvio but Marialva shows similar reductions induced by both treatments. Previous CMS results are therefore correlated with Hélvio’s worse performance when exposed to HST2, but the unexpected differences observed between Celta and Marialva were not envisaged by CMS evaluation.

### *TdHSP101C* nucleotide sequences are highly conserved between varieties

In order to disclose molecular markers useful in the discrimination between heat tolerant genotypes, *TdHSP101C* genes were analyzed in Celta, Hélvio and Marialva varieties. The amplification of *TdHSP101C* coding sequence for the C-terminal region of the corresponding protein was performed using primers previously designed by [21]. In the three genotypes used the PCR reactions yielded a single band which was isolated for sequence analysis (S1 Table). Detailed intra-varietal and inter-varietal comparison of the sequences obtained is presented in Supporting Information (S2 and S3 Tables, respectively). The NCBI BLASTn performed confirmed that the sequences obtained (GenBank KT355875-KT355890) corresponded to the targeted *TdHSP101C* gene region including the AAA+ and ClpB_D2-small conserved domains. *TdHSP101C* gene sequences corresponding to the two known protein forms—form A with 1452bp (chromosome 3A) and form B with 1459bp (chromosome 3B) [20]—were obtained and clearly discriminated. *In silico* analysis of the sequences obtained allowed comparisons at the genomic and peptide levels.

**Fig 2:**
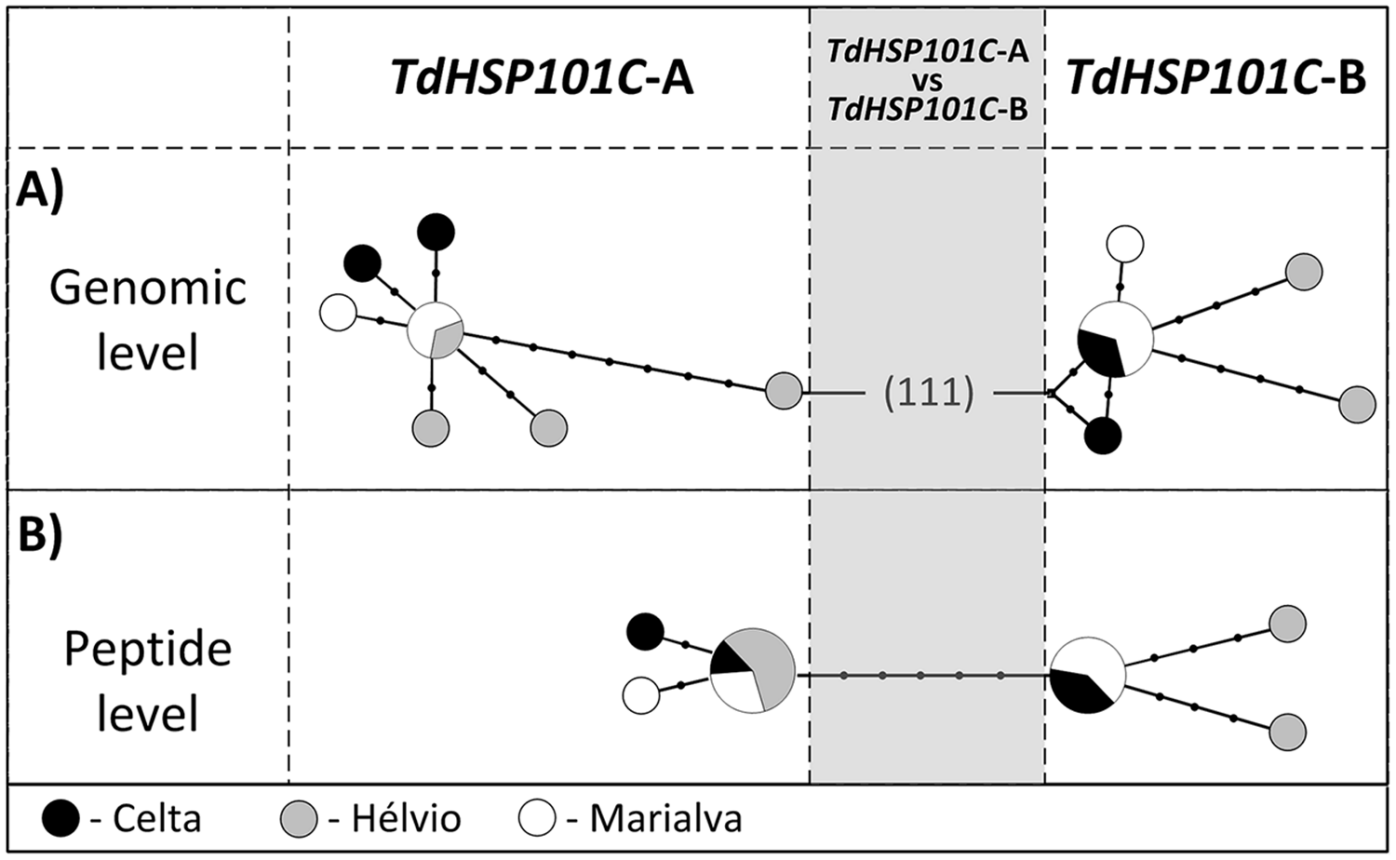
*TdHSP101C* median-joining networks. Median-joining networks for *TdHSP101C*-A and *TdHSP101C*-B genes found in Portuguese durum wheat varieties: Celta (black), Hélvio (grey), Marialva (white). A) Genomic level. B) Peptide level. Grey column shows *TdHSP101C*-A and *TdHSP101C*-B differences between the closest sequences between at genomic and peptide level. Branches are generally proportional to the number of differences between sequences and nodes are proportional to frequencies of sequences. Dots on branches indicate more than one mutational step, except in the grey area at genomic level.

The present comparison between durum wheat *TdHSP101C* sequences revealed high levels of homology (>99% of similarity) and was further used to construct the median-joining network (Fig 2). Differences between the known A and B *TdHSP101C* gene sequences are represented by two distinct groups separated by 111 mutations (Fig 2A). Interestingly, the two commonest sequences of predicted proteins are distinguished only by five conserved SNPs, less than 1.5% of the total sequence (Fig 2B). Predicted peptide comparisons revealed low variability for both forms and only three sequences for each form were observed (Fig 2). Moreover, comparisons between the commonest *TdHSP101C*-A and *TdHSP101C*-B with *T*. *durum* sequences published on NCBI databases [20] showed only one different nucleotide each. Thus, the observed genomic or predicted peptide inter-varietal differences does not explain the distinct thermotolerance of the Portuguese varieties.

### Levels of *TdHSP101C* expression are markedly different between varieties with distinct grain yield after heat stress

**Fig 3:**
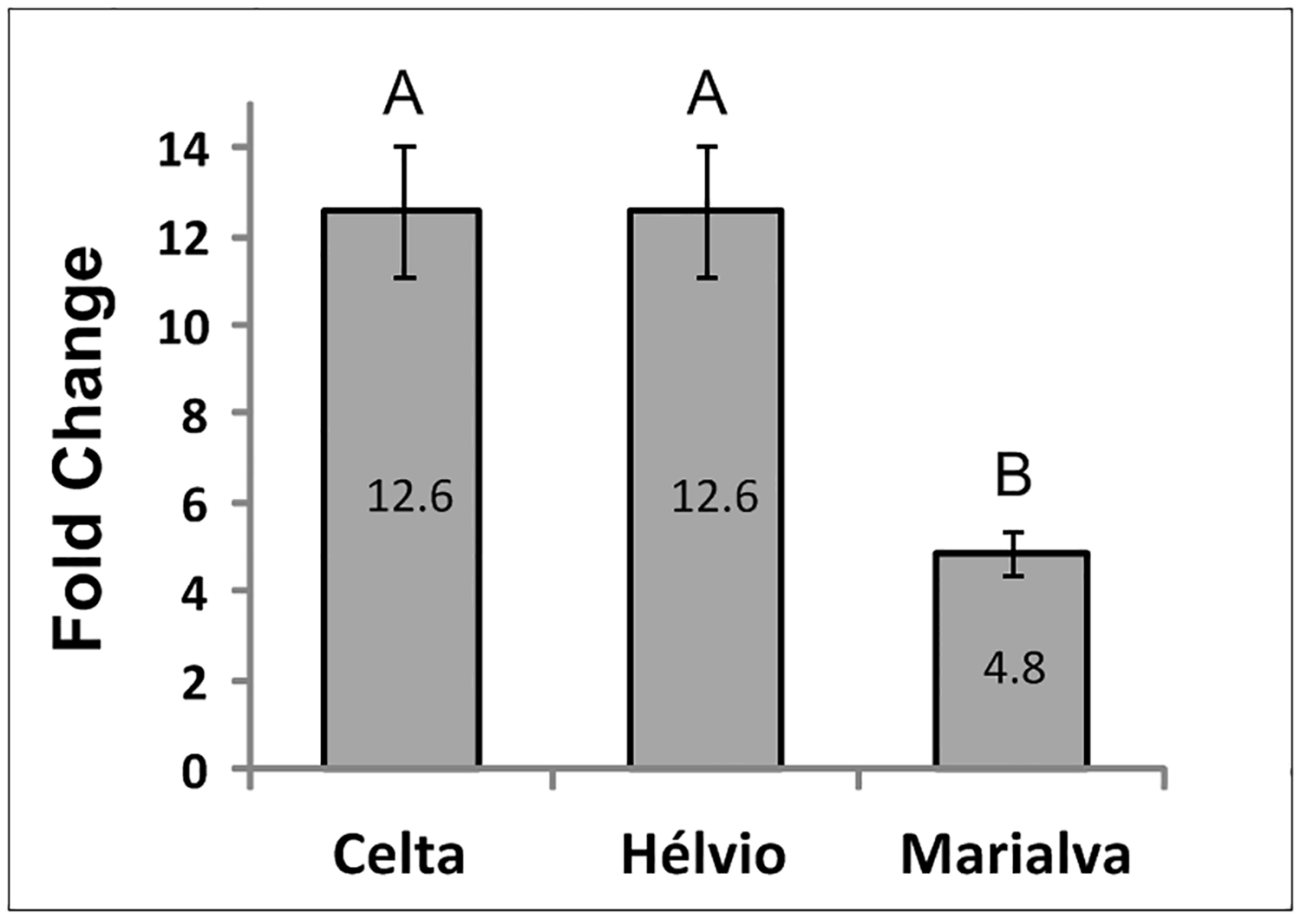
*TdHSP101C* transcription fold change. Comparative analysis of the transcription level between control and high temperature treated plants of each of the three Portuguese durum wheat variety assessed through fold change variation. Means ± SE from three biological replicates. Different letters indicate significant differences (p<0.05) identified by one-way ANOVA.

Due to the residual genomic and peptide differences detected between varieties we further evaluated *TdHSP101C* transcription levels by qRT-PCR using *Actin1* gene as reference. The *TdHSP101C* expression assessment was performed for each variety between control and treatment plants as well as between varieties for both control and treated plants. The differential expression level disclosed by fold change variation between control and heat stressed plants of the same variety (using ΔΔCt = ΔCt stress—mean ΔCt control) unraveled significant differences (p<0.05) between varieties. The *TdHSP101C* transcription increment induced by high temperature correspond to a fold variation of ~12 in Hélvio and Celta varieties while in Marialva that variation was only ~5 (Fig 3).

**Fig 4:**
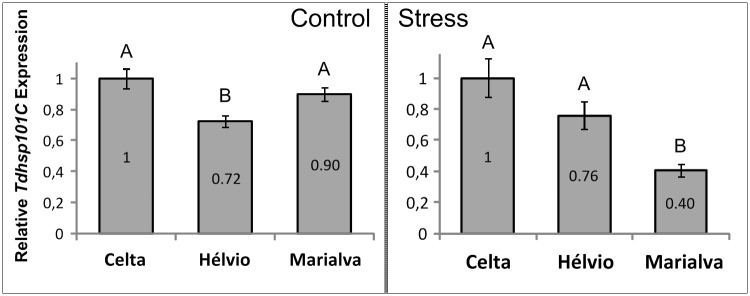
*TdHSP101C* relative transcription levels. *TdHSP101C* transcription levels evaluated in relation to Celta as means ± SE from three biological replicates. Different letters indicate significant differences (p<0.05) identified by one-way ANOVA.

To compare relative expression levels between varieties in control or in high temperature treated plants, Celta mean ΔCt was used as standard and ΔΔCt (ΔΔCt = ΔCt of interest–mean ΔCt Celta) was calculated to estimate mean fold change (2^(-ΔΔCt) ± SE) for Celta, Hélvio and Marialva. In untreated plants, the fold variation of *TdHSP101C* gene expression in comparison to Celta (1±0.06), was 0.90±0.04 for Marialva and 0.72±0.04 for Hélvio (Fig 4, Control) being this last value significantly different (One-way ANOVA, Tukey’s multiple comparison test p<0.05) from those of Celta and Marialva. A similar comparison in heat stress treated plants also revealed significant differences (p<0.05) between the varieties tested, in comparison to Celta (1±0.13) with a fold variation of 0.76±0.09 for Hélvio and 0.40±0.04 for Marialva (Fig 4, Stress). Therefore, the heat treatment effect on transcriptional patterns in early developmental stages is clearly correlated with differential thermotolerance profiles during inflorescence emergence.

## Discussion

The usefulness of cell membrane thermostability (CMS) as a screening method for overall thermotolerance assessment in seedlings of wheat genotypes is well established due to its positive correlation with yield under heat stress in field conditions [9]. CMS has therefore been widely used as an early screening method to select genotypes with contrasting heat stress tolerance profiles [9, 11, 21, 26–28]. Our results also support these observations, since Hélvio variety presented the lowest CMS values and was simultaneously the one that suffered the highest average grain weight reduction (35.7%) when exposed to heat stress during grain filling.

However, we also demonstrate that although CMS is an important methodology for early screening its scope is limited as it cannot predict variations between durum wheat varieties with good CMS results but distinct grain yield outcomes after heat stress. Differences here observed between Celta and Marialva varieties are corroborated by previous results reported in bread wheat where varieties with similar CMS results showed significant yield variations [29]. CMS evaluation is moreover restricted due to the absence of correlations between seedlings thermotolerance values and heat stress impact on yield in plants exposed to stress at different development stages. In the present study we clearly demonstrate such CMS limitations since both Celta and Marialva show good CMS values but differ on heat stress impact on global yield. Such differences seem to depend on the developmental stage affected (Table 1). A significant yield reduction induced by HST1 was observed in one variety with good CMS performance (Marialva with -18% average seed weigh) whereas HST2 induced significant yield reductions in the three varieties tested (variations of 14% in Marialva, 25% in Celta and 36% in Hélvio) (Table 1), being Marialva the less affected variety. The limitation of CMS assessment may result not only from developmental stage disparity but also from differences between the physiological response of detached leaf segments and the whole plant integrated response to stress, as was reported in wheat for the modulation of chlorophyll fluorescence parameters induced by high temperature [30]. The present work new evidences highlighted the need for complementary early screening tools contributing to a clear discrimination between varieties with good CMS performance.

The expression of *HSP*s has been the most studied molecular process to understand plants heat stress responses (reviewed in [31]). In durum wheat the expression of *TdHSP101* genes has been linked to increased thermotolerance and it was suggested a particular involvement of *TdHSP101C* in acquired thermotolerance [20,21]. Thus, we elected *TdHSP101C* genes for genomic and transcriptomic analysis to evaluate their potential usefulness as tools to complement heat tolerance early screening on durum wheat varieties. Our genomic analysis of *TdHSP101C* forms revealed a high level of homology between sequences from Celta, Hélvio and Marialva (>99% of similarity) and most of the detected variability is not translated to the resulting peptide (Fig 2). Notably, the transcriptomic analysis of untreated and heat treated seedlings unraveled a significant correlation with the heat tolerance during inflorescence emergence stage.

In control conditions the two varieties with high CMS values (Celta and Marialva) revealed similar *TdHSP101C* transcript levels (Fig 4, Control) which are however significantly distinct after heat stress exposure (Fig 4, Stress). This difference is due to a higher upregulation of *TdHSP101C* in Celta than Marialva (Fig 3). Such contrasting profiles are very pronounced since levels of *TdHSP101C* transcripts after heat stress are even higher in Hélvio (low CMS values but high fold change) than in Marialva, which can explain its good performance in HST1. The accumulation of HSPs in sensitive organs and tissues is usually interpreted as playing an important role in protection of cell metabolic functions [4]. Particularly, a significant increases in boll set and seed numbers was obtained in transgenic cotton lines were *AtHSP101* is active in pollen when exposed to high temperature [32]. The results here presented seem therefore to corroborate those reports since it can explain why the two varieties with good CMS (Celta and Marialva) have such different yield performances when exposed to HST1 or HST2 treatments. Moreover, the protective importance of *TdHSP101C* seems to be dependent of the plant developmental stage affected by heat stress.

This work disclosed that durum wheat varieties with lower *TdHSP101C* transcription are higher impacted on grain yield suggesting the assessment of *TdHSP101C* expression levels of young expanded leaves as a promising tool to complement CMS screening in the selection durum wheat varieties with superior thermotolerance.

## Supporting information

S1 Table Accession numbers. Accession numbers of *TdHSP101C* coding sequence for the C-terminal region of the corresponding protein of distinct Portuguese durum wheat varieties.
(PDF)

S2 Table *TdHSP101C* intra-varietal comparison. Intra-varietal comparison of *TdHSP101C* partial sequences in Portuguese durum wheat varieties.
(PDF)

S3 Table *TdHSP101C* inter-varietal comparison. Inter-varietal comparison of *TdHSP101C* partial sequences and comparison between the two forms identified in Portuguese durum wheat varieties.
(PDF)
