# Supplementary material for: Durum wheat diversity for heat stress tolerance during inflorescence emergence is correlated to TdHSP101C expression in early developmental stages
Source: PLoS One. 2017 Dec 28;12(12):e0190085. doi: 10.1371/journal.pone.0190085 (PMC5746240; doi:10.1371/journal.pone.0190085)
Supplement: S1 Table — Accession numbers of TdHSP101C coding sequence for the C-terminal region of the corresponding protein of distinct Portuguese durum wheat varieties. (PDF) [file pone.0190085.s001.pdf]

**S1 Table. Accession numbers.** Accession numbers of *TdHSP101C* coding sequence for the C-terminal region of the corresponding protein of distinct Portuguese durum wheat varieties.

| **Accession** | **Definition** |
|---|---|
| KT355875 | Triticum turgidum subsp. durum cultivar Celta haplotype B1 heat shock protein (hsp101C-B) gene, partial cds |
| KT355876 | Triticum turgidum subsp. durum cultivar Celta haplotype B2 heat shock protein (hsp101C-B) gene, partial cds |
| KT355877 | Triticum turgidum subsp. durum cultivar Helvio haplotype B1 heat shock protein (hsp101C-B) gene, partial cds |
| KT355878 | Triticum turgidum subsp. durum cultivar Helvio haplotype B2 heat shock protein (hsp101C-B) gene, partial cds |
| KT355879 | Triticum turgidum subsp. durum cultivar Marialva haplotype B1 heat shock protein (hsp101C-B) gene, partial cds |
| KT355880 | Triticum turgidum subsp. durum cultivar Marialva haplotype B2 heat shock protein (hsp101C-B) gene, partial cds |
| KT355881 | Triticum turgidum subsp. durum cultivar Marialva haplotype B3 heat shock protein (hsp101C-B) gene, partial cds |
| KT355882 | Triticum turgidum subsp. durum cultivar Celta haplotype A1 heat shock protein (hsp101C-A) gene, partial cds |
| KT355883 | Triticum turgidum subsp. durum cultivar Celta haplotype A2 heat shock protein (hsp101C-A) gene, partial cds |
| KT355884 | Triticum turgidum subsp. durum cultivar Helvio haplotype A1 heat shock protein (hsp101C-A) gene, partial cds |
| KT355885 | Triticum turgidum subsp. durum cultivar Helvio haplotype A2 heat shock protein (hsp101C-A) gene, partial cds |
| KT355886 | Triticum turgidum subsp. durum cultivar Helvio haplotype A3 heat shock protein (hsp101C-A) gene, partial cds |
| KT355887 | Triticum turgidum subsp. durum cultivar Helvio haplotype A4 heat shock protein-like (hsp101C-A) gene, partial sequence |
| KT355888 | Triticum turgidum subsp. durum cultivar Marialva haplotype A1 heat shock protein (hsp101C-A) gene, partial cds |
| KT355889 | Triticum turgidum subsp. durum cultivar Marialva haplotype A2 heat shock protein (hsp101C-A) gene, partial cds |
| KT355890 | Triticum turgidum subsp. durum cultivar Marialva haplotype A3 heat shock protein (hsp101C-A) gene, partial cds |
