# Supplementary material for: Durum wheat diversity for heat stress tolerance during inflorescence emergence is correlated to TdHSP101C expression in early developmental stages
Source: PLoS One. 2017 Dec 28;12(12):e0190085. doi: 10.1371/journal.pone.0190085 (PMC5746240; doi:10.1371/journal.pone.0190085)
Supplement: S2 Table — Intra-varietal comparison of TdHSP101C partial sequences in Portuguese durum wheat varieties. (PDF) [file pone.0190085.s002.pdf]

S2 Table. *TdHSP101C* Intra-varietal comparison. Intra-varietal comparison of *TdHSP101C* partial sequences in Portuguese durum wheat varieties.

			Celta		Marialva		Hélvio	
	**Variety**		**Celta**		**Marialva**		**Hélvio**	
	**Total of sequences**		4		6		6	
	Form (no. sequences)		A (2)	B (2)	A (3)	B (3)	A (4)	B (2)
**Genomic level**	Consensus size (bp)		1452	1459	1452	1459	1452	1459
	Similarity (≠s bp)		99.86% (2)	99.93% (1)	99.93% (1)	99.93% (1)	99.37% (9)	99.52% (7)
	Differences (SNPs/Gaps)		2/0	1/0	1/0	1/0	7/2	7/0
**mRNA level**	Consensus size (bp)		1112	1167	1112	1167	1112	1167
	Similarity (≠s bp)		99.82% (2)	100% (0)	99.91% (1)	99.91% (1)	99.18% (9)	99.49% (6)
	Differences (SNPs/Gaps)		2/0	0/0	1/0	1/0	6/2	6/0
**Protein level**	Total ≠ peptides		2	1	2	1	1	2
	Consensus size (bp)		341	341	341	341	341	341
	Similarities (≠s aa)		99.71% (1)	100% (0)	99.71% (1)	100% (0)	100% (0)	98.24% (6)
	Differences (amino acids)	Strongly similar Properties	1	0	0	0	0	0
		Weakly similar Properties	0	0	1	0	0	2
		Dissimilar Properties	0	0	0	0	0	4
