# Supplementary material for: Durum wheat diversity for heat stress tolerance during inflorescence emergence is correlated to TdHSP101C expression in early developmental stages
Source: PLoS One. 2017 Dec 28;12(12):e0190085. doi: 10.1371/journal.pone.0190085 (PMC5746240; doi:10.1371/journal.pone.0190085)
Supplement: S3 Table — Inter-varietal comparison of TdHSP101C partial sequences and comparison between the two forms identified in Portuguese durum wheat varieties. (PDF) [file pone.0190085.s003.pdf]

S3 Table. ***TdHSP101C*** **Inter-varietal comparison.** Inter-varietal comparison of *TdHSP101C* partial sequences and comparison between the two forms identified in Portuguese durum wheat varieties.

	Variety		Celta vs Marialva vs Hélvio		
	***TdHSP101C* form**		**A**	**B**	**TdHSP101C-A vs TdHSP101C-B**
	No. of sequences		2 / 3 / 4	2 / 3 / 2	9 / 7
**Genomic level**	Different sequences		2 / 2 / 4	2 / 2 / 2	7 / 5
	Consensus size (bp)		1452	1459	1469
	Similarity (≠s bp)		99.38% (13)	99.38% (9)	91.01% (131)
	Differences (SNPs/Gaps)		11 / 2	9 / 0	106 / 26
**mRNA level**	Different sequences		2 / 2 / 4	1 / 2 / 2	6 / 4
	Consensus size (bp)		1112	1167	1169
	Similarity (≠s bp)		98.92% (12)	99.91% (9)	90.59% (110)
	Differences (SNPs/Gaps)		10/2	9/0	51 / 59
**Protein level**	≠ peptides per variety		2 / 2 / 1	1 / 1 / 2	-
	Total ≠ peptides		3	3	3 / 3
	Consensus size (bp)		341	341	341
	Similarity (≠s aa)		99.41% (2)	98.24% (6)	95.31% (13)
	Differences (amino acids)	Strongly similar properties	1	0	3
		Weakly similar properties	1	2	5
		Dissimilar properties	0	4	5
